# Incidence of skeletal‐related events in patients with Ewing sarcoma: An observational retrospective study in Japan

**DOI:** 10.1002/cam4.7060

**Published:** 2024-03-11

**Authors:** Hisaki Aiba, Yuki Kojima, Tatsunori Shimoi, Kazuki Sudo, Shu Yazaki, Toru Imai, Akihiko Yoshida, Shintaro Iwata, Eisuke Kobayashi, Akira Kawai, Ayumu Arakawa, Chitose Ogawa, Hiroaki Kimura, Kan Yonemori

**Affiliations:** ^1^ Department of Medical Oncology National Cancer Center Hospital, National Cancer Center Hospital Tokyo Japan; ^2^ Department of Orthopaedic Surgery Nagoya City University Nagoya Japan; ^3^ Department of Diagnostic Pathology National Cancer Center Hospital, National Cancer Center Hospital Tokyo Japan; ^4^ Department of Musculoskeletal Oncology and Rehabilitation National Cancer Center Hospital, National Cancer Center Hospital Tokyo Japan; ^5^ Department of Pediatric Oncology National Cancer Center Hospital Tokyo Japan

**Keywords:** bone metastasis, Ewing sarcoma, metastasis, radiotherapy, skeletal‐related event

## Abstract

**Background:**

Skeletal‐related events (SREs), including the pathological fracture, surgical treatment or radiation of bone lesions, malignant spinal cord compression, hypercalcemia, are important considerations when managing metastatic bone tumors; however, owing to their rarity, the incidence of SREs in patients with Ewing sarcoma remains unknown.

**Methods:**

We retrospectively reviewed the clinical data from 146 patients with Ewing sarcoma treated at a single institution from 2005 to 2019. The median age at diagnosis was 22.7 years. Fifty patients (34.2%) had metastatic disease at diagnosis. The primary outcome was the SRE‐free rate among patients with Ewing sarcoma. Moreover, we identified the risk factors for SREs using univariate or multivariate analyses.

**Results:**

During the observational period (median, 2.6 years), SREs occurred in 23 patients. Radiation to the bone, malignant spinal cord compression, and hypercalcemia were documented as the initial SREs in 12 patients (52.2%), 10 patients (43.5%), and one patient (4.3%), respectively. The SRE‐free rate was 94.2 ± 2.0, 87.3 ± 3.0, and 79.6 ± 3.8% at 1, 2, and 3 years after the initial visit, respectively. Multivariate analysis revealed bone metastasis at diagnosis (hazard ratio [HR] = 4.41, *p* = 0.007), bone marrow invasion (HR = 34.08, *p* < 0.001), and local progression or recurrence after definitive treatment (HR = 3.98, *p* = 0.012) as independent risk factors for SREs.

**Conclusions:**

SREs are non‐rare events that can occur during the treatment course for Ewing sarcoma, with an especially high incidence of malignant spinal cord compression. Patients with metastatic disease at diagnosis, especially in the bone or bone marrow, or with local progression or recurrence after definitive treatment, should be carefully monitored for the occurrence of SREs. The most effective methods to monitor the occurrence of SREs and new preventative therapies for SREs should be investigated in the future.

## 1 INTRODUCTION

Ewing sarcoma (EWS) is the second most common primary bone tumor in children and adolescents.^1^ EWS occurs in approximately 2–3 patients/1,000,000, and distant metastases at diagnosis are reported in approximately 30% of patients.^1^ The most common metastatic sites are the lungs, bones, and bone marrow.^2^

The risk of skeletal‐related events (SREs), including pathological fracture, surgery, radiation to the bone, malignant spinal cord compression (MSCC), and hypercalcemia,^3^ should be carefully considered in the management of bone metastasis, in addition to controlling tumor progression. Approximately 40%–70% of patients with advanced prostate or breast cancer will develop bone metastasis during the course of disease progression.^4^, ^5^, ^6^ A prospective study of 751 stage IV breast cancer patients with bone metastasis revealed that approximately 50%–60% of these patients experienced SREs.^7^ SREs are associated with impaired quality of life and activities of daily living,^4^ as well as heavy burdens for caregivers and society.^6^ A study of the economic burden of solid tumors with bone metastases revealed that patients with SREs incurred an average of $67,257 in additional health‐care costs annually^8^; therefore, focusing on the prediction and prevention of SREs is important.

However, although bone metastasis has been frequently reported in patients with metastatic EWS,^1^ the actual status of SREs in patients with EWS has not been the focus of previous research. In this study, we investigated SREs among patients with EWS to elucidate the frequency and risk factors of SREs.

## 2 METHODS

This retrospective observational/comparative study was performed at a single institution. The patients were treated between January 2005 and December 2019, and their data were collected from the institutional database.

**FIGURE 1:**
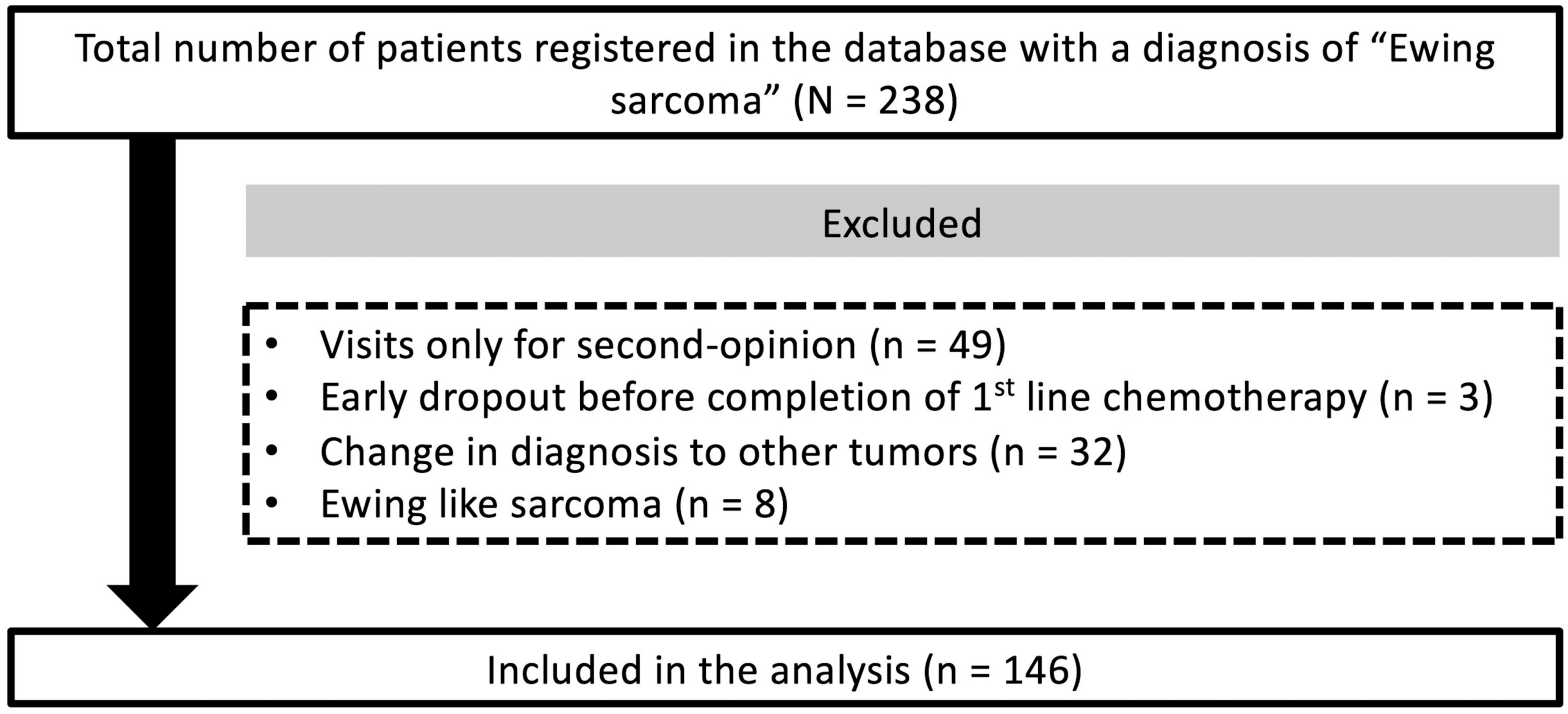
Flowchart of patient selection process. From a total of 238 patients with “Ewing sarcoma” registered in the database, we excluded patients who visited our hospital only for a second opinion (*n* = 49) and had insufficient information due to early dropout before the completion of first‐line treatment (*n* = 3). In addition, 32 patients whose diagnosis changed from “Ewing sarcoma” to “other tumors” and eight patients with Ewing‐like sarcoma were excluded. Eventually, 146 patients with Ewing sarcoma were included in the analysis.

The definitive diagnosis of EWS was based on histological and/or molecular cytogenetic findings (i.e., the presence of hallmark gene mutations).^9^ Among the 238 patients with a provisional diagnosis of “Ewing sarcoma” registered in the database, we excluded patients who visited our hospital only for a second opinion (*n* = 49) and had insufficient information due to early dropout before the completion of first‐line treatment (*n* = 3). In addition, 32 patients with a provisional diagnosis of “Ewing sarcoma” that eventually changed to “other tumors” (e.g., rhabdomyosarcoma, desmoplastic small round cell tumor) and eight patients with a diagnosis of Ewing‐like sarcoma, including *CIC‐DUX4* or *BCOR‐CCNB3* fusion‐related sarcoma, were excluded. Eventually, 146 patients (144 Asian, one Caucasoid, and one African) were included in the analysis (Figure 1). The median follow‐up period was 2.6 years (interquartile range [IQR], 1.3–6.0 years).

### 2.1 Description of treatments and investigations

Evaluation of the primary location and distant metastasis sites was performed before chemotherapy induction using appropriate modalities (computed tomography, magnetic resonance imaging, positron emission tomography/computed tomography [PET/CT]), and spinal fluid or bone marrow specimens were collected (not mandatory).

Treatment procedures were determined via multidisciplinary conferences. The basic treatment strategy included intensive chemotherapy for localized disease (vincristine‐doxorubicin‐cyclophosphamide/ifosfamide‐etoposide [VDC‐IE] administered as a 2‐week regimen [q2w] in pediatric patients and a 3‐week regimen [q3w] in adolescents), followed by definitive surgery with or without radiotherapy.^10^ In pediatric patients with metastasis at diagnosis, high‐dose chemotherapy followed by hematopoietic cell transplantation (HDC/HCT) was considered.^11^ Older patients, or patients unfit for VDC‐IE with metastatic disease, underwent less intensive chemotherapy.^12^

Response Evaluation Criteria in Solid Tumors (RECIST) 1.1 was used to evaluate the primary tumor's response to neoadjuvant therapy. The four response categories included in the RECIST 1.1 were as follows: complete response (CR), the disappearance of all target lesions; partial response (PR), target lesion diameter decreased by >30%; progressive disease (PD), target lesion diameter increased by >20%; and stable disease (SD), a lesion that does not meet the other criteria.^13^

### 2.2 Statistical analysis

The primary outcome of this study was to evaluate the frequency of SREs that occurred in patients with EWS. SREs were defined as fractures, surgery, radiation to the bone, MSCC, and hypercalcemia.^3^ We did not include events or pathologic fractures of the primary lesion, surgery or radiation for primary lesions, and MSCC due to direct invasion from primary spinal EWS as SREs in the analysis. The SRE‐free rates were estimated using the Kaplan–Meier method, defined as the time from the initial visit to the medical institution to the day the first SRE occurred. The timing of SREs by different SRE subcategories is shown via probability density functions. In addition, we calculated the risk factors for SREs using univariate or multivariate analyses via log‐rank or Cox regression analysis as a secondary outcome. Further, we evaluated the relationship between the occurrence of SREs and overall survival (OS), which was estimated using the Kaplan–Meier method as the time from diagnosis to death by any cause. Moreover, we conducted a supplementary analysis in which we assessed the risk factors influencing OS and progression‐free survival (PFS), delineated as the time from diagnosis to either disease progression or death from any cause, with Cox regression analysis. Values were compared using Student's *t*‐test or the Mann–Whitney *U* test along with the distribution of characteristics. The value distributions are presented as means or medians, as appropriate, based on the Shapiro–Wilk analysis. Statistical analysis was performed using SPSS version 26 (IBM Corp, Armonk, NY, USA). Statistical significance was set at *p* < 0.05 (bilateral).

## 3 RESULTS

### 3.1 Patient demographics

Data from 146 patients were analyzed in this study. Overall, 87 men (59.6%) and 59 women (40.4%) were included, with a median age at diagnosis of 22.7 years (IQR, 15.3–33.0). The primary tumor sites were skeletal and extra‐skeletal in 61 (41.8%) and 85 (58.2%) patients, respectively. At diagnosis, 96 patients (65.8%) had localized disease, and 50 presented (34.2%) with metastatic disease. For precise staging, some patients underwent cerebrospinal fluid and bone marrow puncture examinations; the disseminations were observed in cerebrospinal fluid (positive for 2/56 patients [3.6%]) and bone marrow (positive for 6/95 patients [6.3%]) (Table 1).

**TABLE 1:** Characteristics of the patients.

Variables	Subcategory	Value
Age, year (median, IQR)		22.7 (15.3–32.9)
Tumor length, mm (median, IQR)		78 (46–110)
Sex	Men	87 (59.6%)
	Women	59 (40.4%)
ECOG‐PS score at initial visit	0, 1, 2, 3, 4	39, 84, 13, 6, 4 (26.7%, 57.5%, 8.9%, 4.1%, 2.7%)
Primary location (skeletal)	Vertebrae	28 (13.7%)
	Pelvis	17 (11.6%)
	Other bones	15 (10.3%)
Primary location (extra‐skeletal)	HENNT	16 (11.0%)
	Chest wall/thoracic organs	13 (8.9%)
	Peritoneal/retroperitoneal organs	27 (18.5%)
	Soft tissue or skin	30 (20.5%)
Metastasis at diagnosis^a^	Total	50 (34.2%)
	Bone metastasis	22 (15.1%)
	Lymph node	22 (15.1%)
	Cerebrospinal fluid^b^	2 (3.6%)
	Bone marrow^c^	6 (6.3%)

Abbreviations: ECOG‐PS, Eastern Cooperative Oncology Group Performance Status; HENNT, head, ears, eyes, nose, and throat; IQR, interquartile range.

^a^ Distribution of metastasis had overlaps.

^b^ Investigation was conducted on 56 patients.

^c^ Investigation was conducted on 95 patients.

Regarding systemic therapy, a total of 103 patients (70.5%) underwent scheduled (q3w for 17 cycles in 44 patients [30.1%], q2w for 14 cycles in 26 patients [17.8%]) and unscheduled (21 patients with 99%–80% [14.4%], seven patients with 80%–50% [4.8%], and five patients [3.4%] with less than 50% intensity) of the scheduled VDC‐IE therapy. HDC/HCT therapy was administered to patients with metastasis (12 patients, 8.2%). Eighteen patients with metastatic disease (12.3%) received vincristine, actinomycin D, and cyclophosphamide/ VDC therapy to stabilize disease progression. Eleven patients received other regimens (7.5%), and two patients (1.4%) received no chemotherapy.

The change in tumor size was evaluated from the tumor size measured before and after at least four cycles of chemotherapy. Among the 91 patients with targetable lesions, the response to first‐line chemotherapy was evaluated as CR (15 patients, 16.5%), PR (43 patients, 47.3%), SD (30 patients, 33.0%), and PD (3 patients, 3.3%).

Regarding definitive treatments of the primary lesion, 72 patients received a definitive surgery (49.3%), 38 patients received a definitive surgery with radiotherapy (26.0%), 8 patients received a definitive radiotherapy (5.5%), and 28 patients received no definitive therapy (19.2%).

### 3.2 Occurrence of SREs

**FIGURE 2:**
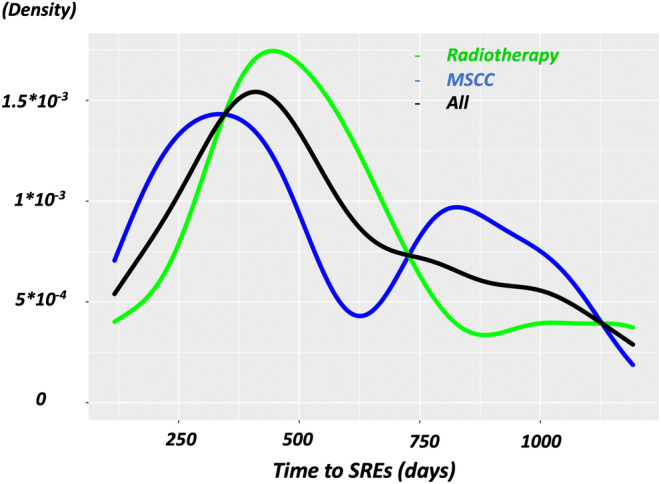
Time course of SREs. The probability density curves show the different time courses of the SREs among radiotherapy (green), MSCC (blue), and all (black) categories. MSCC, malignant spinal cord compression; SRE, skeletal‐related event.

**FIGURE 3:**
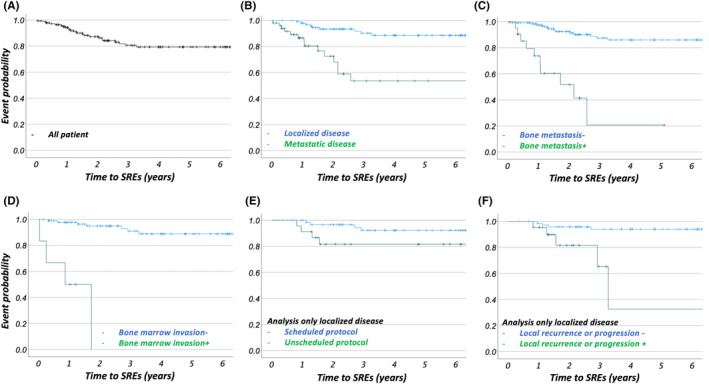
Kaplan–Meier analysis for SRE‐free rate among all patients and by categorized groups. Time courses of SREs among all patients (a) and by categorized groups, highlighting the differences between patients; (b) without metastasis (localized disease) versus with any metastasis at diagnosis (HR = 6.03, *p* < 0.001); (c) without bone metastasis versus with bone metastasis at diagnosis (HR = 9.91, *p* < 0.001); (d) with and without bone marrow invasion at diagnosis (HR = 34.49, *p* < 0.001, only inspected cases were analyzed); (e) with scheduled VDC‐IE (q2w + q3w) versus unscheduled regimens (only patients with localized EWS treated with VDC‐IE therapy were included in the analysis; HR = 0.26, *p* = 0.024); and (f) with or without local recurrence/progression after definitive treatment for localized disease (only patients without metastasis who underwent definitive treatments were included in the analysis; HR = 8.02, *p* = 0.002). EWS, Ewing sarcoma; HR, hazard ratio; q2w, 2‐week regimen: q3w, 3‐week regimen; SREs, skeletal‐related events; VDC‐IE, vincristine‐doxorubicin‐cyclophosphamide/ifosfamide‐etoposide.

During the follow‐up period, SREs were observed in 23 patients, including radiation to the bone in 12 patients (52.2%; 12/23), MSCC from the metastatic site in 10 patients (43.5%; 10/23), and hypercalcemia in one patient (4.3%; 1/23). SREs occurred in 10 patients (43.5%: 10/23) more than two times. The median timing of each event was 459 days (IQR: 334–764 days) for all events, 516 days (IQR: 389–656 days) for patients who received radiotherapy, and 362 days (IQR: 206–826 days) for patients with MSCC. Figure 2 shows the overall timing of the SREs with the probability density functions. Kaplan–Meier analysis revealed that the SRE‐free rates were 94.2 ± 2.0, 87.3 ± 3.0, and 79.6 ± 3.8% at 1, 2, and 3 years after the initial visit, respectively (Figure 3A).

The occurrence of SREs in different primary lesions is shown in Table S1 and Figure S1. Significant differences were observed between the incidence of SREs in the pelvis versus soft‐tissue/skin lesions (*p* = 0.005) and the pelvis versus visceral organs (*p* = 0.042).

We examined the oncologic outcome after SREs and found that the median time from the day of the SRE to death from any cause was 158 days (95% confidence interval [CI], 116–200; Figure S2).

### 3.3 Risk factors for SREs

Univariate analysis indicated the primary lesion site (skeletal vs extra‐skeletal; hazard ratio [HR] = 2.43, 95% CI [1.09–5.42], *p* = 0.027), presence of metastasis at diagnosis (HR = 6.03, 95% CI [2.71–10.45], *p* < 0.001), bone metastasis at diagnosis (HR = 9.91. 95% CI [4.38–22.41], *p* < 0.001), and bone marrow invasion at diagnosis (HR = 34.49, 95% CI [8.76–135.78], *p* < 0.001) as risk factors for SREs. Regarding localized disease, the local recurrence or progression events after definitive treatments were associated with SRE occurrence (HR = 8.02, 95% CI [2.12–30.31], *p* = 0.002); the intensity of chemotherapy (scheduled vs less intensity, HR = 0.26, 95% CI [0.08–0.83], *p* = 0.024) was also associated with SREs. The response to induction therapy did not affect the incidence of SREs (SD‐PD vs. PR‐CR, HR = 1.20, 95% CI [0.93–1.56], *p* = 0.16). Figure 3B–F shows the SRE‐free rate of the selected categories.

Following multivariate analysis, metastasis at any sites (HR = 3.26, 95% CI [1.31–8.11], *p* = 0.011), presence of bone metastasis at diagnosis (HR = 4.41, 95% CI [1.51–12.91], *p* = 0.007), bone marrow invasion at diagnosis (HR = 34.1, 95% CI [8.66–134.14], *p* < 0.001), and local recurrence or progression events after definitive treatments (HR = 3.98, 95% CI [1.36–11.63], *p* = 0.012) were identified as independent risk factors for SREs (Table 2).

**TABLE 2:** Univariate or multivariate analysis of SRE occurrence.

Variables	Univariate analysis HR (95% CI)	*p*‐Value	Multivariate analysis HR (95% CI)	*p*‐Value
Age (>18 years vs. ≤18yeas [ref])	1.37 (0.61–3.07)	0.45		
Sex (men vs. women [ref])	0.80 (0.37–1.72)	0.56		
ECOG‐PS score (2–4 vs. 0–1 [ref])	2.01 (0.75–5.34)	0.16		
Primary location (skeletal vs. extra‐skeletal [ref])	2.43 (1.09–5.42)	0.027		
Metastasis to any location (yes vs. no [ref])	6.03 (2.71–10.45)	<0.001	3.26 (1.31–8.11)	0.011
Bone metastasis (yes vs. no [ref])	9.91 (4.38–22.41)	<0.001	4.41 (1.51–12.91)	0.007
Lymph node metastasis (yes vs. no [ref])	1.85 (0.69–4.96)	0.22		
Bone marrow invasion (yes vs. no [ref])	34.49 (8.76–135.78)	<0.001	34.1 (8.66–134.14)	<0.001
Response to first‐line chemotherapy (SD‐PD vs. PR‐CR [ref])	1.20 (0.93–1.56)	0.16		
Intensity of chemotherapy (scheduled vs. less intensity [ref])^a^	0.26 (0.08–0.83)	0.024		
Occurrence of the local recurrence or progression (yes vs. no [ref])	8.02 (2.12–30.31)	0.002	3.98 (1.36–11.63)	0.012

Abbreviations: CI, confidence interval; CR, complete response; ECOG‐PS, Eastern Cooperative Oncology Group Performance Status; HR, hazard ratio; PD, progressive disease; PR, partial response; ref, reference; SD, stable disease; SRE, skeletal‐related event.

^a^ Analysis included patients with localized Ewing sarcoma treated with vincristine‐doxorubicin‐cyclophosphamide/ifosfamide‐etoposide therapy (64 and 23 patients with scheduled and low‐intensity regimens, respectively).

Moreover, supplementary information detailing the risk factors associated with additional oncologic outcomes, including OS and PFS, can be found in Tables S2 and S3.

### 3.4 Effect of SREs on oncologic outcomes

We investigated whether the occurrence of SREs affected oncologic outcomes. Figure S3 shows the OS rates of patients with or without SREs. Based on the univariate analysis, patients with SRE had worse OS among all the patients (HRs = 4.21, 95% CI, 2.52–7.03, *p* < 0.001), patients with localized disease at diagnosis (HR = 6.22, 95% CI, 2.82–13.74, *p* < 0.001), and patients with metastatic disease at diagnosis (HR = 1.63, 95% CI, 0.84–3.19, *p* = 0.152).

## 4 DISCUSSION

Bone metastasis is a common event in patients with advanced solid cancers, leading to SREs and deterioration of quality of life.^14^ To prevent SREs, especially MSCC, clinicians should be aware of the subtle preceding symptoms and carefully monitor any changes in imaging findings^15^; the initial symptoms of SREs include newly worsening pain, weakness or numbness of the extremities, band‐like sensation on the trunk, and bladder or rectal disturbance.^15^ Here, we reviewed the occurrence of SREs among patients with EWS and revealed that these events were not rare; the cumulative incidence of SREs was approximately 20% at 3 years after the initial visit.

We further found that common SREs included radiotherapy for painful bones (52.2%) or MSCC (43.5%). The markedly higher incidence of MSCC in patients with EWS differed from the SREs that occurred in patients with other solid cancers.^13^ Hong et al. studied SREs using a national Korean database and analyzed 21,562 newly diagnosed patients with various types of cancers and 1849 patients with bone metastases and reported that the most common SRE was radiation therapy to the bone (70.5%), followed by bone surgery (8.4%), and MSCC (3.4%).^14^ The reasons for the different patterns of SREs between patients with EWS versus other solid tumors are unclear; however, the high affinity to the bone as the metastatic site and the unique ability to rapidly spread to extra‐skeletal lesions through the Haversian canal without destroying the bony lesion may be related to this difference.^16^

Regarding the treatment of bone metastasis of EWS, a study from the Memorial Sloan Kettering Cancer Center showed that a 3‐year local control rate of bone metastasis by 50 Gy of radiotherapy of 91%.^17^ However, for patients with multifocal metastases (>5), radiotherapy is not considered an effective method for tumor regulation.^17^ Moreover, data from 120 patients registered in the European Ewing Tumor Working Initiative of National Groups (EURO‐E.W.I.N.G. 99) trial demonstrated the importance of local therapy for bone metastasis of EWS.^18^, ^19^ According to their report, long‐term survivors with metastatic disease only included patients who underwent local radiotherapy for bone metastasis (3‐year‐event‐free survival = 39% vs. 13% in the radiotherapy and nonradiotherapy groups, respectively).^18^, ^19^ Based on these findings, radiotherapy should be considered in patients with bone single‐oligometastases before the possible occurrence of SREs.

Moreover, regarding pharmacotherapy for bone metastasis, several bone‐modulating agents are expected to effectively prevent SREs via modulation of osteoclast activity.^20^ Experimentally, although EWS cells cannot destroy bone, they induce osteoclast activation and subsequent bone resorption (a well‐known vicious cycle in the bone microenvironment).^21^, ^22^ However, the efficacy of zoledronic acid (ZOL), a bone‐modulating agent, has not been proven. The Ewing 2008R1 phase III, prospective, randomized controlled clinical trial (EudraCT2008‐003658‐13) was conducted to evaluate the effect of ZOL as maintenance therapy for patients with standard risk EWS.^20^ The researchers compared the additional effects of nine cycles of maintenance ZOL after standard induction therapy in 284 patients.^20^ However, event‐free survival rates were not significantly different between the ZOL and control groups (HR = 0.74, 95% CI: 0.43–1.28).^19^ Thus, they concluded that for patients with standard risk, localized EWS, maintenance treatment with ZOL provides no benefit; an increased incidence of side effects was also observed in patients receiving ZOL treatment.^20^ Moreover, to date, the efficacy of denosumab for regulating EWS has not yet been proven.

This study reported that bone marrow invasion at the initial diagnosis was the strongest risk factor for SREs (HR = 34.1). Approximately 5% of patients with all newly diagnosed EWS and less than 20% with metastatic disease reportedly present with bone marrow metastasis.^23^ According to the 2022‐National Comprehensive Cancer Network guideline for initial staging workups, bone marrow biopsy should be considered. However, a recent meta‐analysis stated that PET/CT might be a substitute for bone marrow biopsy due to its excellent detection potential.^24^ A report of the analysis of 504 patients with EWS revealed that 12 (2.4%) patients had bone marrow invasion and 91.6% (11/12) had simultaneous bone metastasis; thus, a strong relationship between bone metastasis and bone marrow involvement was suggested.^25^ However, in this study, multivariate analyses revealed that bone marrow invasion at diagnosis (HR = 34.1) was more strongly related to the occurrence of SREs than bone metastasis at diagnosis (HR = 4.41), indicating that bone marrow biopsy is important in the initial workup.

Local recurrence or progression after definitive treatments were related to the occurrence of SREs according to the univariate (HR = 8.02) and multivariate (HR = 3.98) analyses, which show the association between the difficulty of local control and the occurrence of SREs. Indeed, considering that the occurrence of SREs at 3 years was approximately 40% in the pelvis and 10% of soft tissue origin, some correlations were observed between the resectability of the primary tumor and the SREs. However, visceral organs, which are also in a difficult position for local treatment, had a moderate occurrence rate of SREs (approximately 20% at 3 years). This difference might be related to the genetic characteristics of EWS and bone tropism in skeletal EWS.^16^ Gene ontology analysis revealed that skeletal system development and extracellular matrix genes were highly expressed in the skeletal EWS population.^26^ Disease progression due to differences in gene expression has not yet been elucidated.

This study has some limitations. First, this study was performed in a single institution. The EWS distribution in the database was not balanced compared with the general EWS distribution because our institution is a cancer/sarcoma center in Japan that treats EWS of rare origins, which might have influenced our results. Second, during the observation periods, the emergence of a new modality for the treatment (e.g., proton/heavy ion beam therapy, surgical navigation system, and new implantations of the bone defects), development of the inspection equipment, and transition of the guidelines of the systemic therapy might have influenced the study results. In addition, with the development of an accurate EWS classification system, the subset group has emerged as a new entity with different clinical characteristics. We excluded *CIC‐DUX4* and *BCOR‐CCNB3* fusion‐related sarcoma owing to the different behaviors of the new entity.^27^ However, we did not exclude these new entities and other rare variants of the EWS family before 2015. Finally, our findings unveiled some overlaps in risk factors between inferior PFS and the occurrence of SREs. However, SREs demonstrated a higher HR in factors associated with the bone involvement, such as bone metastasis at diagnosis (HR = 4.41, not significant, for SREs, PFS, respectively) or bone marrow invasion (HR = 34.1, 3.22, for SREs, PFS, respectively), underscoring the imperative need for increased vigilance in monitoring and addressing the occurrence of SREs in patients with these risk factors.

In conclusion, although bone is considered a predilection site for metastases in patients with EWS, no studies to date have focused on SREs in patients with EWS. We found that SREs are non‐rare events in the course of EWS treatment. The characteristics of SREs in patients with EWS may differ from those of other cancers in that MSCC, the most serious SRE, occurred frequently. Although we observed that patients with metastatic disease at diagnosis, especially to the bone or bone marrow, or with local progression or recurrence after definitive treatment were at risk of SREs, the most effective method to monitor for the occurrence of SREs and identify preventative therapies should be examined in future studies.

## AUTHOR CONTRIBUTIONS

**Hisaki Aiba:** Conceptualization (lead); data curation (lead); formal analysis (lead); investigation (lead); methodology (lead); project administration (lead); software (lead); validation (equal); visualization (lead); writing – original draft (lead); writing – review and editing (equal). **Yuki Kojima:** Conceptualization (supporting); data curation (supporting); investigation (supporting); project administration (supporting); supervision (supporting); writing – review and editing (supporting). **Tatsunori Shimoi:** Conceptualization (supporting); data curation (supporting); project administration (supporting); supervision (supporting); writing – review and editing (supporting). **Kazuki Sudo:** Project administration (supporting); supervision (supporting); writing – review and editing (supporting). **Shu Yazaki:** Project administration (supporting); writing – review and editing (supporting). **Toru Imai:** Investigation (supporting); project administration (supporting); writing – review and editing (supporting). **Akihiko Yoshida:** Methodology (supporting); project administration (supporting); resources (supporting); supervision (supporting); writing – review and editing (supporting). **Shintaro Iwata:** Supervision (supporting); writing – review and editing (supporting). **Eisuke Kobayashi:** Supervision (supporting); writing – review and editing (supporting). **Akira Kawai:** Resources (supporting); supervision (supporting); writing – review and editing (supporting). **Ayumu Arakawa:** Supervision (supporting); writing – review and editing (supporting). **Chitose Ogawa:** Resources (supporting); supervision (supporting); writing – review and editing (supporting). **Hiroaki Kimura:** Supervision (supporting); writing – review and editing (supporting). **Kan Yonemori:** Conceptualization (supporting); project administration (lead); resources (lead); supervision (lead); writing – original draft (supporting); writing – review and editing (supporting).

## FUNDING INFORMATION

This study received no financial support.

## CONFLICT OF INTEREST STATEMENT

Kan Yonemori participates in the advisory boards of Novartis, Eisai, AstraZeneca, Chugai, Takeda, Genmab, and OncXerna; gains corporate‐sponsored research support from MSD, Daiichi‐Sankyo, AstraZeneca, Taiho, Pfizer, Novartis, Takeda, Chugai, Ono, Sanofi, Seattle Genetics, Eisai, Eli Lilly, Genmab, Boehringer Ingelheim, Kyowa Hakko Kirin, Nihon Kayaku, and Haihe. In addition, Kan Yonemori received honorarium for lectures from Pfizer, Eisai, AstraZeneca, Eli Lilly, Takeda, Chugai, Fuji Film Pharma, MSD, Boehringer Ingelheim, Ono, Daiichi‐Sankyo, and Sanofi.

## ETHICS STATEMENT

This study was approved by the ethical committee of the National Cancer Center Hospital (approval number: 2012‐335) and conducted in accordance with the Declaration of Helsinki.

## PATIENT CONSENT

The requirement for informed consent was waived by the ethical committee owing to the retrospective nature of the study and use anonymized data.

## PERMISSION TO REPRODUCE MATERIAL FROM OTHER SOURCES

Not applicable.

## Supporting information


Figure S1.

Figure S2.

Figure S3.

Table S1.

Table S2.

Table S3.

## Data Availability

The data that support the findings of this study are available on request from the corresponding author. The data are not publicly available due to privacy or ethical restrictions.
