# Supplementary figures and images for: Incidence of skeletal‐related events in patients with Ewing sarcoma: An observational retrospective study in Japan
Source: Cancer Med. 2024 Mar 11;13(5):e7060. doi: 10.1002/cam4.7060 (PMC10926881; doi:10.1002/cam4.7060)

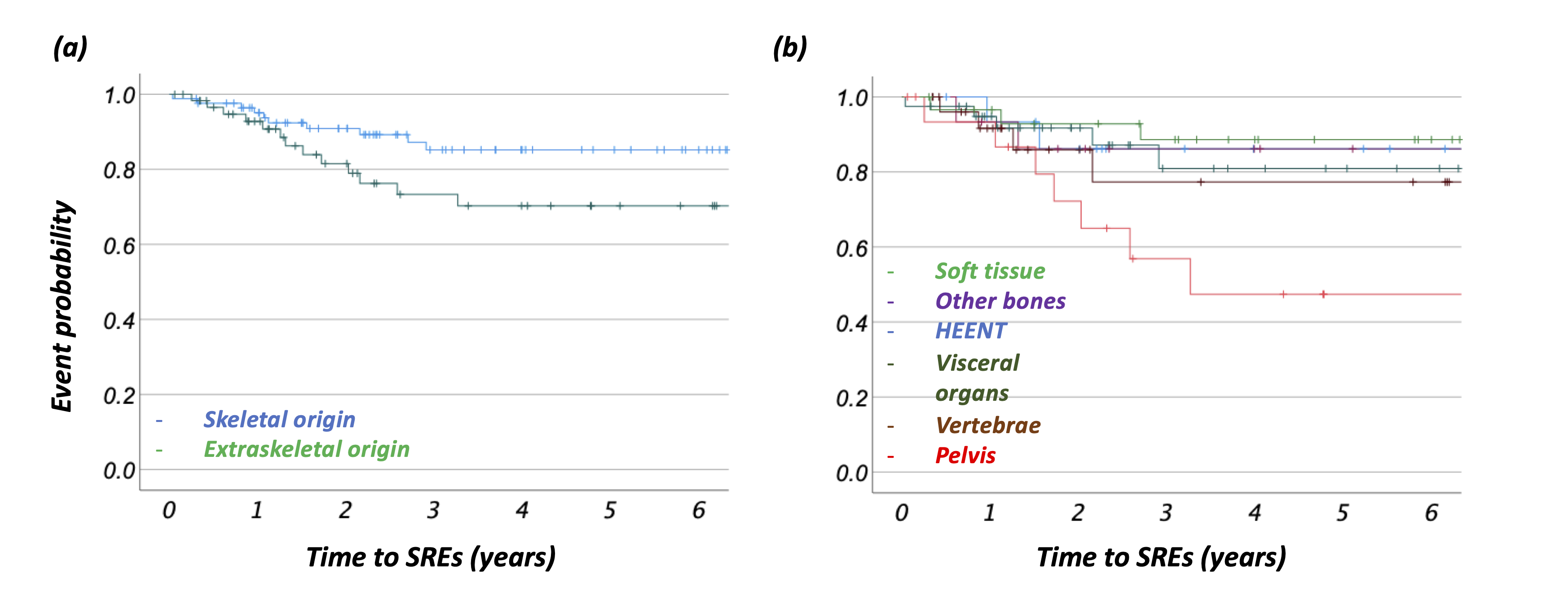

Supplement: Supplementary file 1 — Figure S1. [file CAM4-13-e7060-s004.tiff]

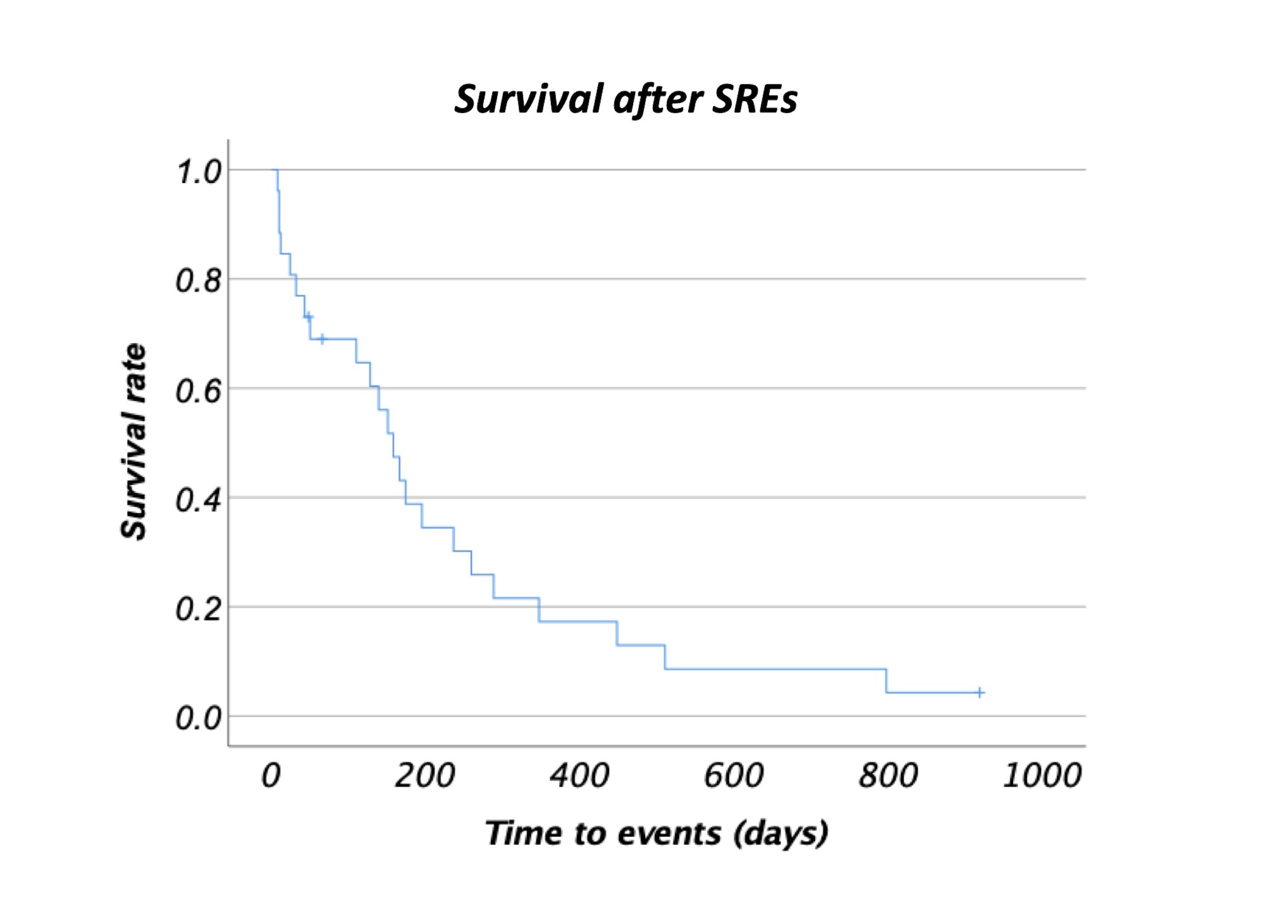

Supplement: Supplementary file 2 — Figure S2. [file CAM4-13-e7060-s002.jpg]

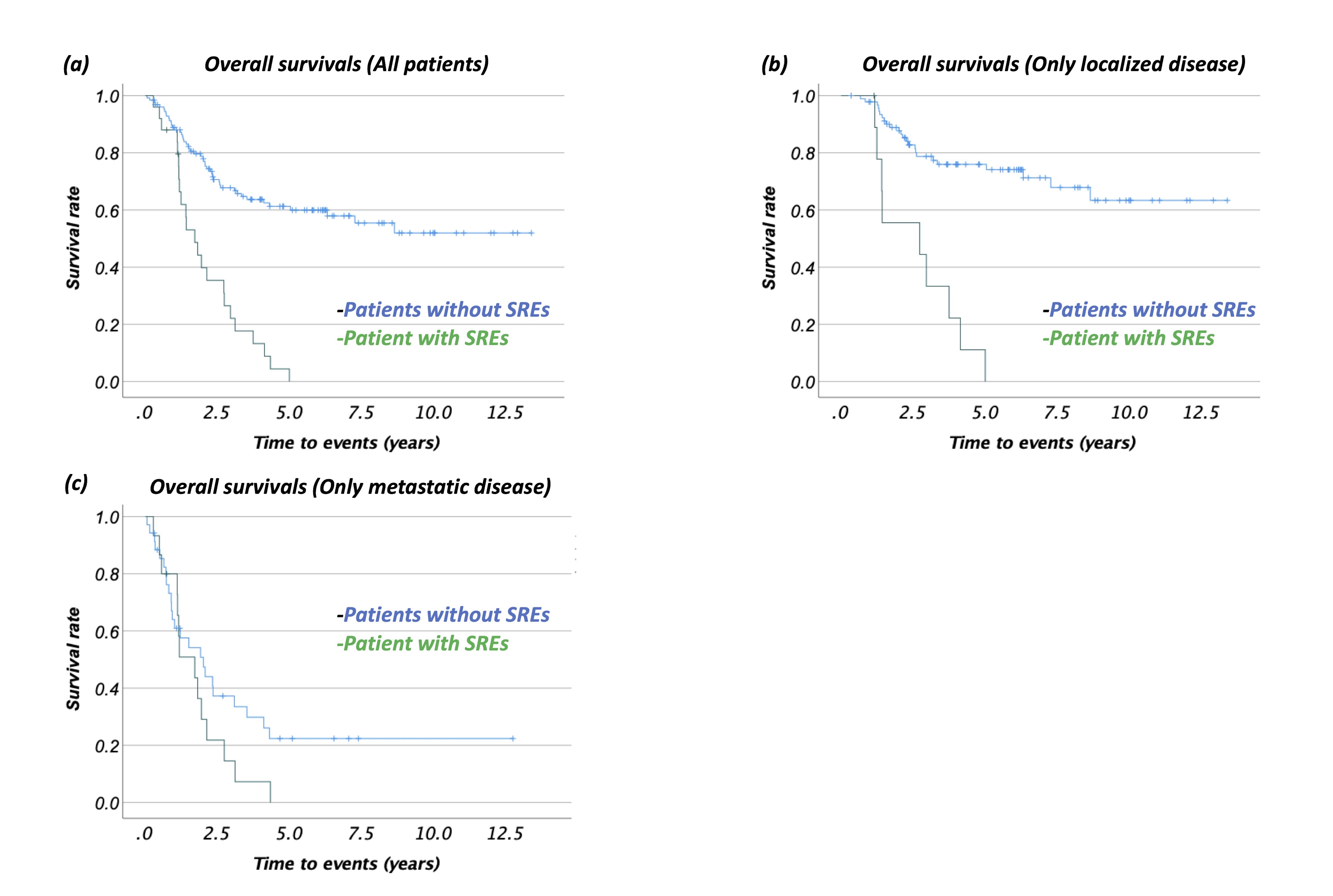

Supplement: Supplementary file 3 — Figure S3. [file CAM4-13-e7060-s001.jpg]
