# Supplementary material for: Incidence of skeletal‐related events in patients with Ewing sarcoma: An observational retrospective study in Japan
Source: Cancer Med. 2024 Mar 11;13(5):e7060. doi: 10.1002/cam4.7060 (PMC10926881; doi:10.1002/cam4.7060)
Supplement: Supplementary file 4 — Table S1. [file CAM4-13-e7060-s003.docx]

**Supporting Information**

**Supplemental Table 1. SRE occurrence in different primary lesions**

| Position | Number | SREs-free rate at 1 year | SREs-free rate at 2 years | SREs-free rate at 3 years |
| --- | --- | --- | --- | --- |
| **Skeletal** | **60** | **92.8% (±3.5)** | **81.5% (±5.6)** | **73.3% (±6.8)** |
| Vertebrae | 28 | 91.6% (±5.7) | 85.9% (±7.7) | 77.3% (±10.7) |
| Pelvis | 17 | 93.3% (±6.4) | 72.2% (±11.9) | 56.9% (±14.2) |
| Other bones | 15 | 93.3% (±6.4) | 86.2% (±9.1) | 86.2% (±9.1) |
| **Extraskeletal** | **86** | **95.1% (±2.4)** | **90.9% (±3.1)** | **85.2% (±4.5)** |
| Soft tissue/skin | 30 | 97.1% (±2.9) | 93.8% (±4.2) | 90.9% (±5.5) |
| HEENT | 16 | 93.3% (±6.4) | 86.2% (±9.1) | 86.2% (±9.1) |
| Visceral organs^†^ | 40 | 94.8% (±3.6) | 91.7% (±4.6) | 80.9% (±8.0) |

^†^Includes the thoracic, peritoneal, and retroperitoneal organs.

HENNT, head, ears, eyes, nose, and throat; SREs, skeletal related-events
