# Supplementary material for: Incidence of skeletal‐related events in patients with Ewing sarcoma: An observational retrospective study in Japan
Source: Cancer Med. 2024 Mar 11;13(5):e7060. doi: 10.1002/cam4.7060 (PMC10926881; doi:10.1002/cam4.7060)
Supplement: Supplementary file 5 — Table S2. [file CAM4-13-e7060-s005.docx]

**Supplemental table 2. Univariate or multivariate analysis for the risk factor of overall survival**

| Variables | Univariate analysis HR (95% CI) | p-value | Multivariate analysis HR (95% CI) | p-value |
| --- | --- | --- | --- | --- |
| Age (> 18 years vs. ≤ 18yeas [ref]) | 1.29  (0.61–3.07) | 0.21 |  |  |
| Sex (men vs. women [ref]) | 0.71  (0.43–1.16) | 0.17 |  |  |
| ECOG-PS score (2–4 vs. 0–1 [ref]) | 1.85  (1.01–3.39) | 0.047 |  |  |
| Primary location (skeletal vs. extraskeletal [ref]) | 1.34  (0.84–2.16) | 0.223 |  |  |
| Metastasis to any location (yes vs. no [ref]) | 6.44  (1.34–30.99) | 0.002 | 5.0  (1.89–13.23) | 0.001 |
| Bone metastasis (yes vs. no [ref]) | 2.39  (1.15–4.97) | 0.019 | 2.53  (0.75-8.51 | 0.133 |
| Lymph node metastasis (yes vs. no [ref]) | 1.75  (0.69–4.41) | 0.237 |  |  |
| Bone marrow invasion (yes vs. no [ref]) | 4.48  (1.41–14.22) | 0.011 | 3.02  (0.96–9.58) | 0.06 |
| Response to first-line chemotherapy (SD-PD vs. PR-CR [ref]) | 3.37  (1.1–10.32) | 0.034 |  |  |
| Intensity of chemotherapy (scheduled vs. less intensity [ref])^†^ | 0.35  (0.27–0.88) | 0.092 |  |  |
| Occurrence of the local recurrence or progression (yes vs. no [ref]) | 2.05  (1.13–3.7) | 0.017 | 2.08  (1.28-3.34) | 0.003 |

^†^Analysis included patients with localized Ewing sarcoma treated with vincristine-doxorubicin-cyclophosphamide/ifosfamide-etoposide therapy (64 and 23 patients with scheduled and low-intensity regimens, respectively).

CI, confidence interval; CR, complete response; ECOG-PS, Eastern Cooperative Oncology Group Performance Status; HR, hazard ratio; PD, progressive disease; PR, partial response; ref, reference; SD, stable disease.
