# Supplementary material for: Incidence of skeletal‐related events in patients with Ewing sarcoma: An observational retrospective study in Japan
Source: Cancer Med. 2024 Mar 11;13(5):e7060. doi: 10.1002/cam4.7060 (PMC10926881; doi:10.1002/cam4.7060)
Supplement: Supplementary file 6 — Table S3. [file CAM4-13-e7060-s006.docx]

**Supplemental table 3. Univariate or multivariate analysis for the risk factor of progression free survival**

| Variables | Univariate analysis HR (95% CI) | p-value | Multivariate analysis HR (95% CI) | p-value |
| --- | --- | --- | --- | --- |
| Age (> 18 years vs. ≤ 18yeas [ref]) | 3.1  (1.06–9.1) | 0.039 |  |  |
| Sex (men vs. women [ref]) | 0.4  (0.12–1.36) | 0.14 |  |  |
| ECOG-PS score (2–4 vs. 0–1 [ref]) | 5.64  (2.64–11.99) | <0.001 | 5.27  (2.52-11.09) | <0.001 |
| Primary location (skeletal vs. extraskeletal [ref]) | 1.96  (0.73-5.27) | 0.184 |  |  |
| Metastasis to any location (yes vs. no [ref]) | 4.33  (2.79–6.71) | <0.001 | 6.86  (3.6–13.08) | <0.001 |
| Bone metastasis (yes vs. no [ref]) | 4.53  (2.72–7.57) | <0.001 |  |  |
| Lymph node metastasis (yes vs. no [ref]) | 2.54  (0.91-7.06) | 0.074 |  |  |
| Bone marrow invasion (yes vs. no [ref]) | 7.47  (1.27-43.0) | 0.026 | 3.22  (1.25-8.3) | 0.015 |
| Response to first-line chemotherapy (SD-PD vs. PR-CR [ref]) | 2.59  (0.9-7.48) | 0.077 |  |  |
| Intensity of chemotherapy (scheduled vs. less intensity [ref])^†^ | 0.32  (0.1–0.97) | 0.043 | 0.38  0.15-0.96 | 0.041 |

^†^Analysis included patients with localized Ewing sarcoma treated with vincristine-doxorubicin-cyclophosphamide/ifosfamide-etoposide therapy (64 and 23 patients with scheduled and low-intensity regimens, respectively).

CI, confidence interval; CR, complete response; ECOG-PS, Eastern Cooperative Oncology Group Performance Status; HR, hazard ratio; PD, progressive disease; PR, partial response; ref, reference; SD, stable disease.
